# Maternal healthcare utilisation, women empowerment, and delivery care: geographical variations in India

**DOI:** 10.1007/s43999-025-00063-3

**Published:** 2025-05-07

**Authors:** Prachi Verma, Ningombam Sanjib Meitei, Sanjram Premjit Khanganba

**Affiliations:** 1 Human Factors & Applied Cognition Lab, Indian Institute of Technology Indore, Indore, 453552 India; 2 Discipline of Psychology, Indian Institute of Technology Indore, Indore, 453552 India; 3 Ningombam Angouton Memorial Trust, Imphal East, 795008 India; 4 Department of Biosciences and Biomedical Engineering, Indian Institute of Technology Indore, Indore, 453552 India; 5 Center for Electric Vehicles and Intelligent Transport Systems , Indian Institute of Technology Indore, Indore, 453552 India; 6 Centre of Futuristic Defense and Space Technologies , Indian Institute of Technology Indore, Indore, 453552 India

**Keywords:** Healthcare system, Maternal health, Delivery care, Women empowerment, Maternal healthcare utilisation

## Abstract

This study utilises the National Family Health Survey- 5 (NHFS-5) data to compare performance in three key indicators called pillars of maternal health, namely–Maternal Healthcare Utilisation (MHU), Women Empowerment (WE), and Delivery Care (DC) across six zones of India: East, West, North, South, Central, and Northeast. It employs the Statistical Performance Index (SPI) by the World Bank to calculate zonal scores for the pillars. Univariate and multivariate statistical analyses reveal significant zonal disparities in all the three pillars (MHU: *p* < .001, WE: *p* < .002, and DC: *p* < .010). Northeast zone has the lowest MHU score (*M* = 56.52) and the second-lowest DC score (*M* = 46.60), despite having the second highest WE score (*M* = 66.37), only behind the South zone which leads in all pillars (MHU; *M* = 80.38, WE; *M* = 69.21, and DC; *M* = 57.60). WE accounts for only a small part of the variability in MHU (*R*^*2*^= .166), indicating that WE alone is insufficient to improve MHU outcomes. This study emphasises the need for further exploration of factors such as difficult terrains and low hospital density, especially in the Northeast zone.

**Supplementary Information:**

The online version contains supplementary material available at 10.1007/s43999-025-00063-3.

## Introduction

Maternal health is not just a personal concern; it is a fundamental pillar of public health, shaping the well-being of future generations. According to the World Health Organisation (WHO), maternal health refers to the state of women’s health during their pregnancies, deliveries, and postpartum periods [1]. It is vital for both women’s and children’s well-being. To improve the maternal health, reducing maternal mortality is a key priority for public health initiatives globally [2]. In alignment with WHO’s recommendation to overall improve maternal health, urging countries worldwide to reduce maternal mortality by 2030 [3], the Indian government is also enhancing maternal health through initiatives under the National Health Mission (NHM). Key programs like Janani Suraksha Yojana (JSY) and Janani Shishu Suraksha Karyakaram (JSSK), Accredited Social Health Activists (ASHA) play a crucial role in improving access to antenatal and postnatal care. These programs aim to enhance maternal healthcare utilisation (MHU) and reduce mortality by strengthening public health infrastructure [4, 5].

In this study, (a) MHU refers to pregnant women’s accessibility to maternal healthcare services, (b) women empowerment (WE) refers to women’s socio-economic autonomy and accessibility to hygiene, and (c) delivery care (DC) refers to the availability of quality delivery services for pregnant women. Despite various schemes, disparities in healthcare access persist across states in India [6]. India, along with Nigeria, the Democratic Republic of the Congo, Ethiopia, and Pakistan, is among the top five countries with low maternal healthcare service uptake [7–9]. Factors such as WE and the quality of DC hinder MHU [10, 11].

While previous studies have focused on individual-level determinants such as maternal age, education, birth order, wealth quintile, religion, caste, and media exposure [3, 12], as well as women’s autonomy [4], and socioeconomic status [10] of maternal health, they have primarily examined individual-level determinants rather than the interplay of these factors, the integrated interaction of the determinants (MHU, WE, and DC) to improve maternal health outcomes is underexplored. Addressing this gap is important for identifying strategies to improve maternal healthcare. Therefore, this study uniquely analyses the interplay of these determinants using NFHS data by incorporating a wide range of indicators related to antenatal care, postnatal care, women’s decision-making power, economic empowerment, institutional births, and skilled birth attendance. By assessing their performance across different zones—East, West, North, South, Central, and Northeast of India—this study offers a macro-perspective on regional disparities which has not been previously reported in the literature. Furthermore, the inclusion of variables such as menstrual hygiene management and access to mobile phones provides novel insights into previously underexplored dimensions of WE in the context of maternal healthcare.

Studying the interplay of MHU, WE, and DC provides an understanding of maternal health outcomes in India as these three factors are interconnected and influence each other. Effective MHU, which includes antenatal care, postnatal care, and skilled birth attendance, is crucial for reducing maternal and infant mortality. However, access to and utilisation of these services are influenced by WE. Empowered women are more likely to make informed decisions about their health, seek appropriate medical care, and navigate barriers to access [4]. DC encompassing the availability and quality of delivery services, further mediates the impact of MHU and WE, determining whether women receive adequate care during childbirth. By analysing the interdependencies of these three pillars, this study offers a holistic perspective on the factors driving maternal health disparities across different zones in India.

By analysing zones rather than individual states, the study aims to highlight zonal strengths and weaknesses with respect to the pillars. This approach leverages the findings for cooperative endeavours and zonal council’s aim of resource sharing among states for balanced development. Established under the States Reorganisation Act of 1956, Zonal Councils enhance regional cooperation and coordination [13] by addressing Centre-State and inter-State issues through discussions and consultations [14]. They facilitating regional planning, resource sharing, and infrastructure development projects that transcend state boundaries, while maintaining a national perspective. Zonal analysis groups together states that share some common characteristics such as geographical location, economic structure, and cultural similarities [13]. This can help to create more homogenous groupings compared to individual states. This approach can help identify macro-level patterns that may be obscured by individual state analyses. This method allows for a broader regional development. Highlighting zonal disparities fosters inter-state cooperation, enabling knowledge exchange and collective efforts to address common challenges. While state-level policies remain essential, zonal analysis complements them by providing a broader framework to address regional challenges related to MHU, WE, and DC.

Furthermore, by employing the World Bank’s Statistical Performance Index (SPI) [15], this study provides a standardised metric for comparing performance across different regions, enabling the integration of multiple indicators into a single score and providing a comprehensive assessment of each pillar. This approach offers a novel lens for assessing regional strengths and weaknesses, facilitating more targeted and effective healthcare policies. Understanding zonal performances provides valuable insights for researchers and policymakers, aiding national efforts towards a resilient healthcare system. Policymakers can utilise this data to design targeted interventions, such as improving WE in underperforming zones or enhancing MHU infrastructure where needed. This zonal approach ensures more relevant and effective healthcare policies, ultimately improving public health outcomes across India.

## Method

### Framework

The National Family Health Survey- 5 (NFHS- 5), conducted by India’s Ministry of Health and Family Welfare (MoHFW) between 2019 and 2021, is the latest in a series of large-scale, nationally representative surveys. Utilising a multistage, stratified sampling design, covering urban and rural households across all states and union territories (UTs). The NFHS- 5 (2019 - 2021) is the latest edition factsheet [16] and report [17] used for the study. The factsheet is accessible at https://data.gov.in/catalog/national-family-health-survey-nfhs-5 while the report is available at https://main.mohfw.gov.in/sites/default/files/NFHS-5_Phase-II_0.pdf [18, 19]. The NFHS- 5 report includes data collected for 131 indicators based on background characteristics of 74 regions (e.g., u_Assam: urban region of Assam) belonging to 27 states, 8 UTs, and national levels. For example, the detailed health-related data collected on topics such as maternal health (e.g., mothers who had an antenatal check-up in the first trimester), empowerment of women (e.g., currently married women who usually participate in three household decisions), and delivery related indicators (e.g., home births that were conducted by skilled health personnel). The regions are classified into six zones: East, West, North, South, Central, and Northeast. Appendix A in Table 3 gives detailed classification of all 72 regions into six zones. NFHS provides critical insights for evidence-based policymaking and program planning in India.

This study focuses on performance of a zone for a particular pillar of maternal health where a pillar refers to broad categories or overarching themes indicative of the combined performance of several key indicators. Three pillars are defined namely, MHU, WE, and DC based on 22 indicators. Appendix B in Table 4 details the pillars. The dataset offers variables pertinent to these pillars, making it an ideal source for analysis and generating insights. The NFHS dataset’s nationwide, comprehensive coverage ensures reliable data for in-depth analysis, helping identify trends, challenges, and design effective public health interventions across India’s six zones.

### Data processing, statistical analyses, and software tools

From the NFHS- 5 data, the investigators have considered 22 indicators grouped into three pillars, addressing missing values by treating them as zeros and only considering the total numbers of counts (excluding cases treated as zero) as denominator in SPI calculation. To explore the performance rankings of the zones, the investigators adopted a measure, SPI. Using the SPI, the study enables comparative analysis across regions and time, aiming to inform better-targeted policies and resource allocation [15]. The SPI provides a structured, transparent, and stable metric, ensuring equal contribution of all indicators. The SPI’s standardised unique scores allow for direct comparisons across zones, making it more practical for benchmarking and policy-driven decision-making. Given the study’s goal of assessing regional maternal healthcare disparities, SPI is preferred as it offers clear, actionable insights, making it easier to identify underperforming zones and guide targeted interventions. However, a potential disadvantage of using composite indicators like SPI is that they provide a summary measure of performance at a specific point in time, lacking the ability to capture dynamic changes over time. Since SPI combines multiple factors into a single score, it may overlook short-term fluctuations, policy changes, or improvements that occur gradually. This limitation makes it harder to track real-time progress or respond promptly to emerging issues in data systems. Additionally, the aggregation process in SPI may mask specific areas of concern within individual indicators, potentially leading to oversimplified conclusions.

In this study, SPI is applied to calculate performance of a region of a state or a UT, e.g., u_Assam (urban region of Assam) for the pillars MHU, WE, and DC. This index facilitates a structured assessment of the data, ensuring a comprehensive evaluation of pillars in a region by providing a standardised scoring mechanism. In this study the investigators consider a particular case of the original SPI where each dimension is an individual indicator within its pillar, contributing to the overall assessment of that pillar. For detail see Appendix A in Table 3.

Within a particular pillar a score is computed for all the dimensions, which is an unweighted average of the indicators within that dimension if not explicitly specified. Notably, in the current study, each indicator is considered a dimension with equal weighting.

$${SPI.DIM}_{ctpd}={\sum }_{i=1}^{{N}_{I}}\frac{{SPI.IND}_{ctpdi}}{{N}_{I}}$$

SPI.IND_ctpdi_ is an indicator i in dimension d, pillar p, time t (NFHS- 5), and region (r_Assam, u_Assam etc.) c, and N_I_ is the number of indicators in dimension d (here, N_I_ = 1) hence, SPI.DIM_ctpd_ = SPI.IND_ctpdi._

The score for each pillar (SPI.PIL_ctp_) is calculated as follows, where ω_pd_ is the weight for dimension d in pillar p, and N_d_ is the number of dimensions in pillar p (Online Resources 1, 2, and 3).

$${SPI.PIL}_{ctp}={\sum }_{d=1}^{{N}_{d}}\frac{{{\upomega }_{pd} \times SPI.DIM}_{ctpd}}{{N}_{d}}$$

Since each dimension has the same weight ω_pd_ = 1 for all the values of p and d hence,

$${SPI.PIL}_{ctp}={\sum }_{d=1}^{{N}_{d}}\frac{{SPI.DIM}_{ctpd}}{{N}_{d}}$$

Finally, the SPI.PIL_ctp_ scores are used to calculate the quantile and classify the regions into four quantiles (Online Resources 1, 2, and 3). The basic diagnostic insight about the zonal performances concerning the three pillars was arrived at by calculating favourability scores for all the zones, defined as follows.

$$Favourability\; Score (y,z)=\frac{Number\; of\; regions\; with\; Q4\; and\; Q3\; scores\; in\; a\; Zone}{Total\; number\; of\; regions\; in\; the\; Zone}\times 100$$

Where, y = MHU, WE, and DC; z = E, W, N, S, C, and NE; Q4 = Fourth Quantile of SPI.PIL_ctp_; and Q3 = Third Quantile of SPI.PIL_ctp_.

A multivariate analysis of variance (MANOVA) was performed to investigate if there were significant zonal variations in terms of the performance scores for the pillars followed by Bonferroni post-hoc test to bring out the zones with varying performances using SPSS version 27.0.1. The null hypothesis (H₀) for the MANOVA comparing the pillars (MHU, WE, and DC scores) across the six zones of India (East, West, North, South, Central, and Northeast) is:

H₀: There are no statistically significant differences among the means of MHU, WE, and DC scores across the six zones of India (East, West, North, South, Central, and Northeast).

**Fig. 1:**
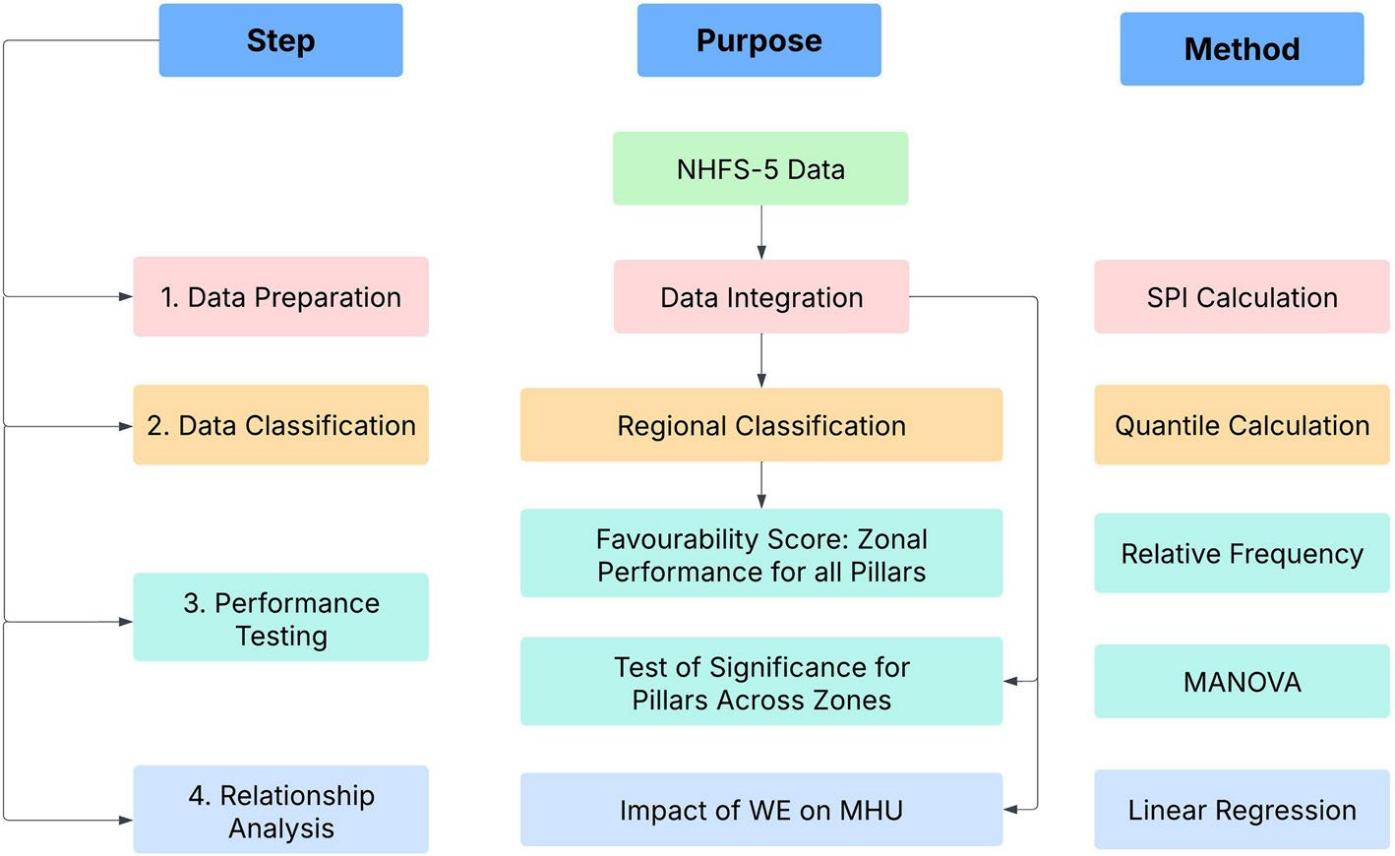
Data processing framework

The investigators examined the relationship between MHU and WE by performing a univariate regression analysis. A visualisation of the relationship was achieved using scatterplot (Microsoft Excel) highlighting the impact of WE on the MHU (i.e., $MHU=WE\times {b}+ a$, where, *a* is the intercept and *b* is the gradient of the linear equation) Overall data processing framework explains step, purpose, and method utilised (Fig. 1).

## Results

### Regional performances based on SPI scores

**Fig. 2:**
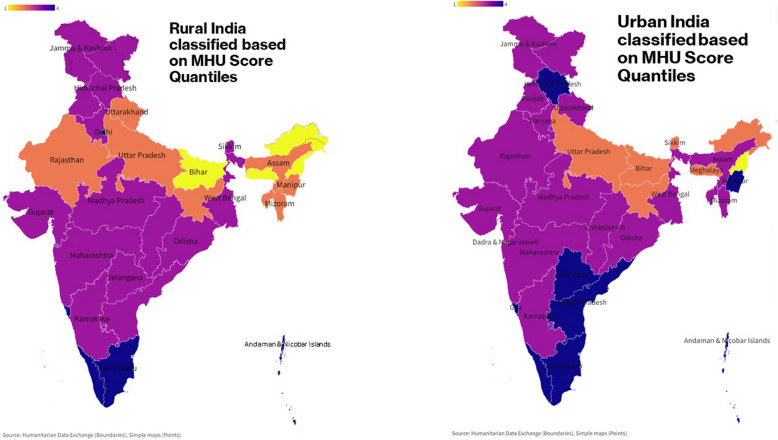
Quantile classification of regions based on SPI scores of MHU

**Fig. 3:**
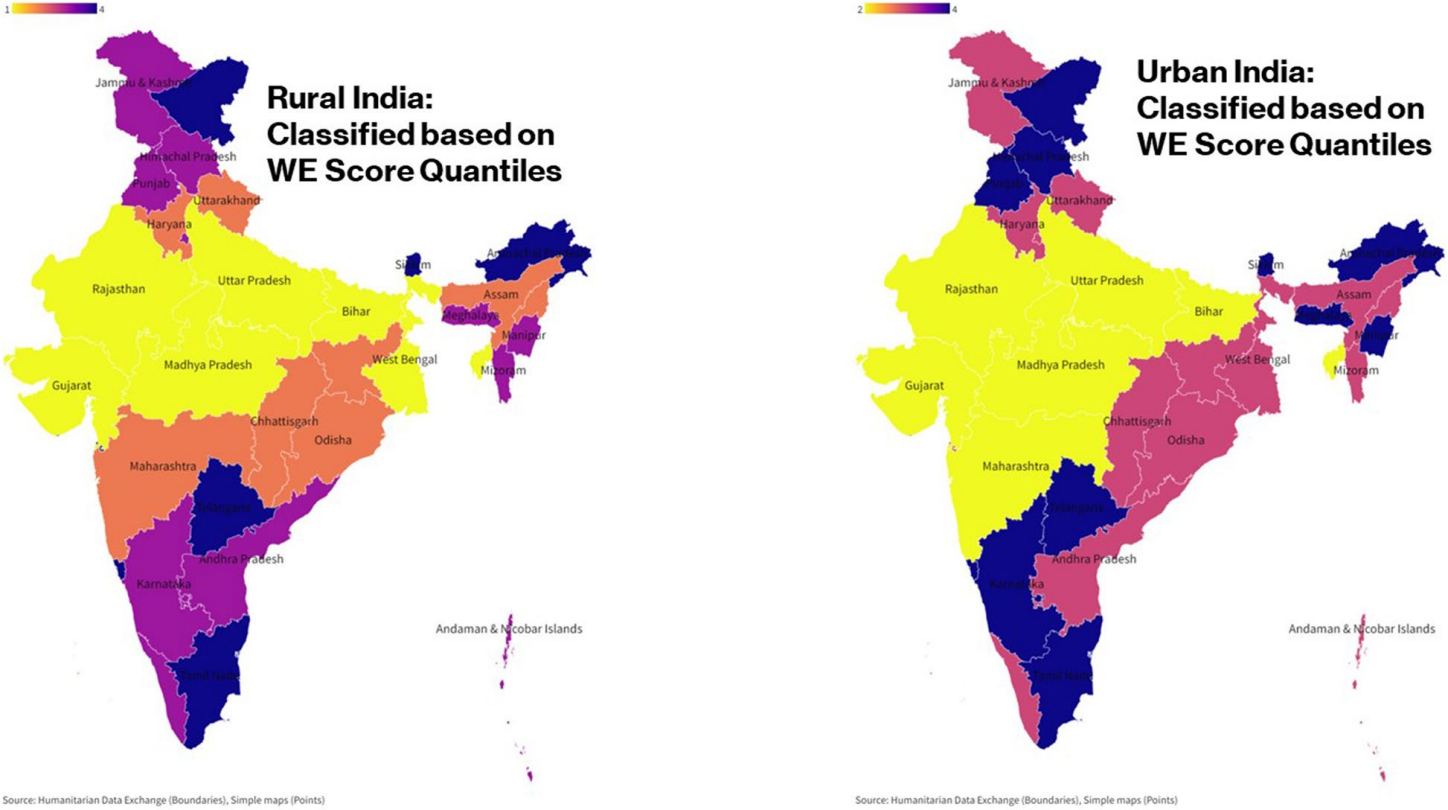
Quantile classification of regions based on SPI scores of WE

**Fig. 4:**
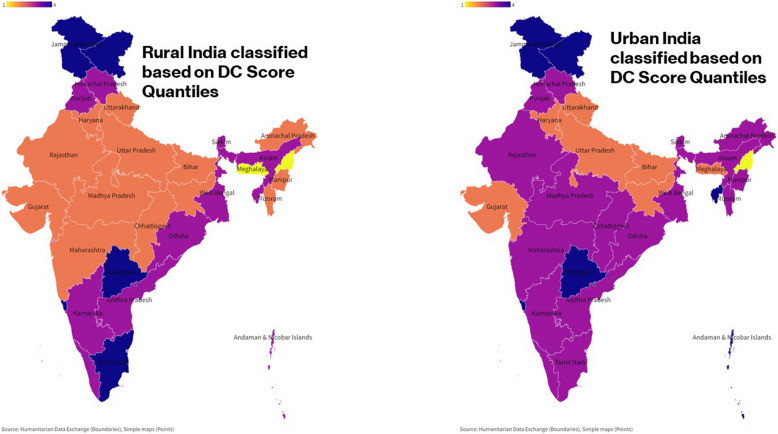
Quantile classification of regions based on SPI scores of DC

The regions are classified into four quantiles based on quantile scores obtained from *SPI.PIl*_*ctp*_ score of the pillars. Quantile 4 represents the excellent performance followed by quantile 3 indicating good performance, quantile 2 indicating fair performance, and quantile 1 indicating poor performance of zones in a pillar. For MHU, 26.76% of the nation fall in the fourth quantile, 46.48% in the third quantile, 19.71% in the second quantile, and 7.04% in the first quantile. For WE, 28.17% are in the fourth quantile, 39.44% in the third quantile, 22.54% in the second quantile, and 9.86% in the first quantile. Finally, for DC, 19.71% fall in the fourth quantile, 45.07% in the third quantile, 30.99% in the second quantile, and 4.22% in the first quantile (Figs. 2, 3, and 4). All the maps presented in this study were generated by the investigators using Flourish platform (https://flourish.studio/visualisations/maps/) [20].

### Zonal favourability scores

**Fig. 5:**
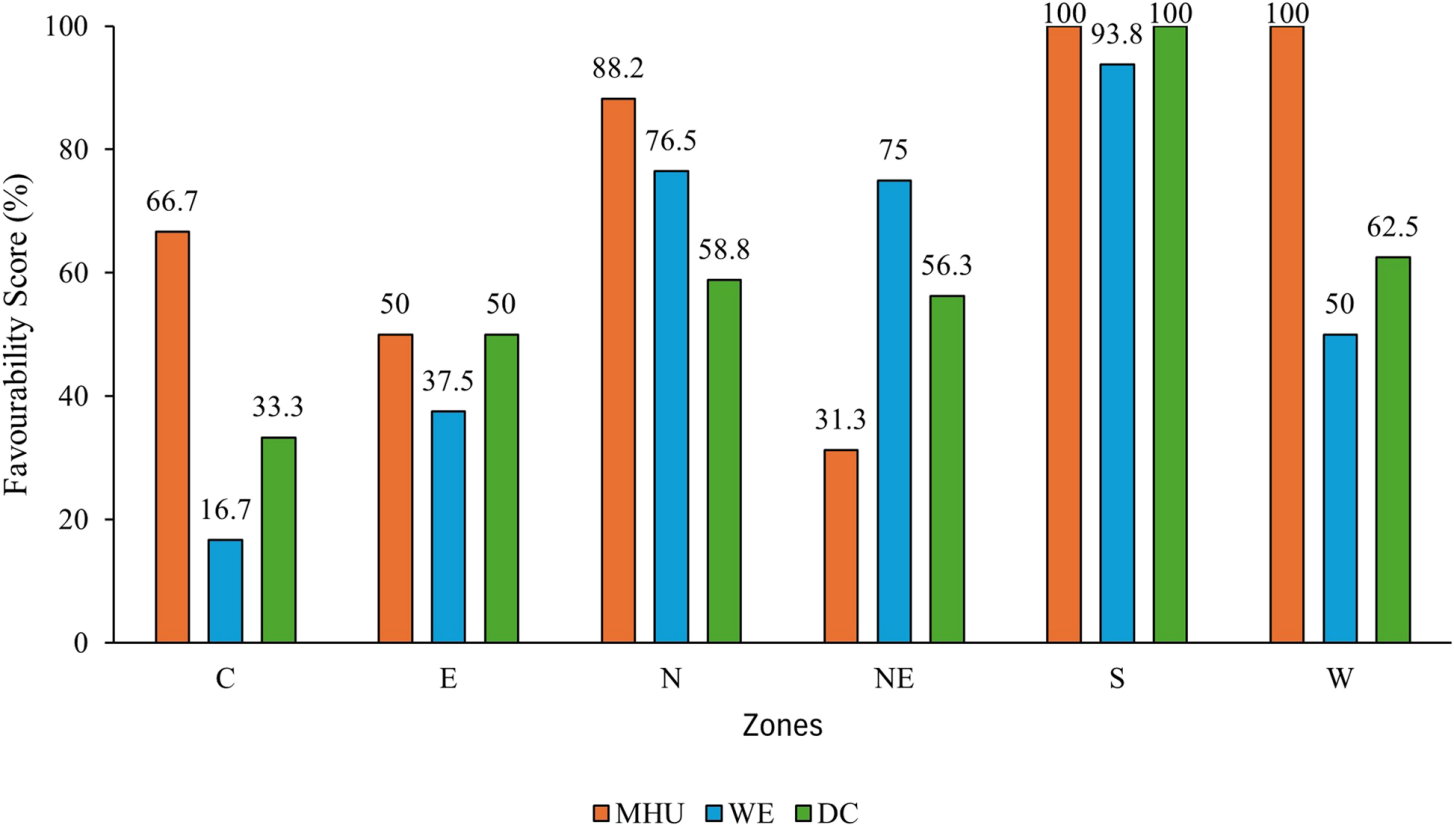
Favourability score of zones based SPI score. Note: C= Central zone, E= East Zone, N= North zone, NE= Northeast zone, S= South zone, W= West zone

As shown in Table 1, for the pillar MHU, West and South achieved a 100% score. At the second rank, North zone scored 88.2%. At the third rank, Central zone scored 66.7%, followed by East zone (50%). The least scoring zone was Northeast, with a score of only 31.3%. For the pillar WE, the South zone scored highest with 93.8%, followed by North zone with a score of 76.5%. Northeast zone scored the third rank with a 75% score, West zone scored 50%, East zone scored 37.5%, and the least scoring zone was Central with a score of 16.7%. For the pillar DC, the South zone scored highest with 100%. The second rank was obtained by West zone with a score of 62.5%, followed by the North zone with a score of 58.8%. Northeast zone score 56.3%. E zone scored 50% while Central zones performed the worst with scoring 33.3% ( Fig. 5).

**Table 1:** Favourability of zones: classification of regions in four quantiles

Zones	Total Regions in the zone	Number of regions with Q4 and Q3 scores in a Zone for the Pillar			Favourability Score (%)		
		MHU	WE	DC	MHU	WE	DC
C	6	4	1	2	66.7	16.7	33.3
E	8	4	3	4	50	37.5	50
N	17	15	13	10	88.2	76.5	58.8
NE	16	5	12	9	31.3	75	56.3
S	16	16	15	16	100	93.8	100
W	8	8	4	5	100	50	62.5

The MANOVA results revealed significant differences across the zones for all three pillars, as indicated by the following *F*-values and *p*-values: MHU: *F* (5, 65) = 15.463, *p* <.001, WE: *F* (5, 65) = 4.175, *p* <.002, and DC: *F* (5, 65) = 3.316, *p* <.010. In the post-hoc comparisons several significant mean differences were found (Table 2).

**Table 2:** Bonferroni multiple comparisons post hoc test for pillars

Zones		MHU	WE	DC
Central	South	− 19.41*	− 9.34*	
	West	− 14.57*		
East	South	− 20.82**	− 7.91*	
	West	− 15.98*		
North	South	− 10.99*		
Northeast	North	− 12.85**		
	South	− 23.85**		− 10.99*
	West	− 19.01**		

Each cell contains Mean Difference between the Zones, specified in the first column, comparing zones specified in the second column for a particular key indicator

^*^p <.05

^**^p <.001

For MHU, both Central and East zone performed worse than both South and West zone; N zone performed worse than South zone; and Northeast zone performed worse than North, South, and West zones. For WE, both Central and East zones performed worse than South zone. For DC, Northeast zone performed worse than South zone (Online Resources 4 and 5).

### Linear regression: impact of WE on MHU

**Fig. 6:**
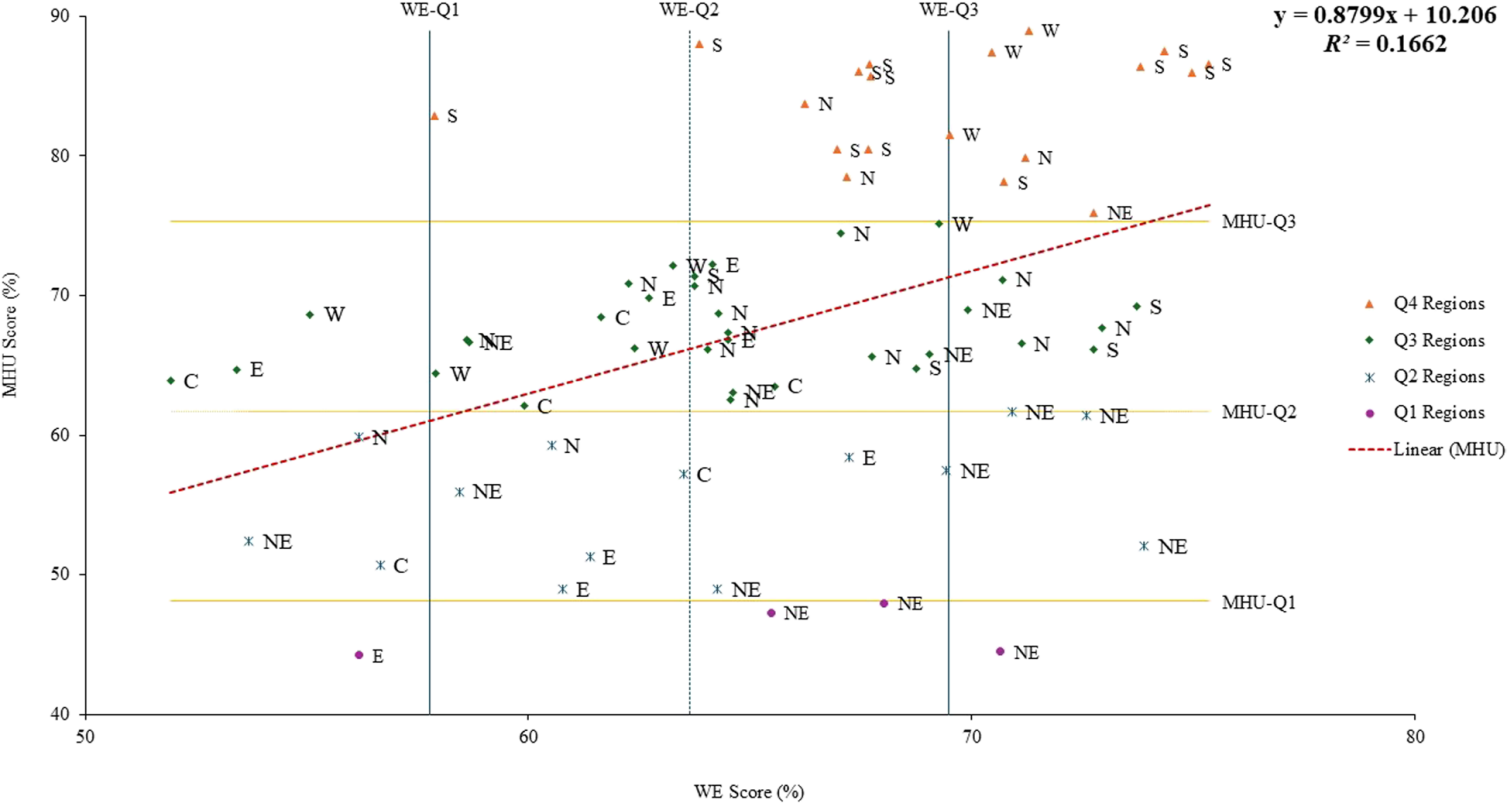
Scatter plot of WE and MHU. Note: C= Central zone, E= East Zone, N= North zone, NE= Northeast zone, S= South zone, W= West zone

Figure 6 shows the scatterplot of WE and MHU showing no linear correlation between WE and MHU with the coefficient of determination (*R*^*2*^) =.166 (Online Resources 6).

## Discussion

### Summary of results

This study emphasises on understanding the performance of zones for the pillars–MHU, WE, and DC. As per the results of the present study, Central zone performed worst in both WE and DC, whereas South zone exceled in all three pillars. Northeast zone exhibits the lowest MHU despite having the second highest WE score. Past researchers have found that empowered women are more likely to seek appropriate medical care and make informed health decisions [4, 19], but the present study indicates that WE alone explains only a small proportion of variance in MHU. Under a particular zone, the states falling within its cluster may share similarities and at the same time may vary in terms of their policies. The zonal disparity analysis of the current study provides unique insight. Zonal councils provide an excellent collective forum where public health issues, that affects multiple states such as regional health disparities amongst states, can be resolved by promoting the inter-state sharing of resources within the zone. The zonal councils are regional platforms of cooperative endeavour for states linked with each other economically, politically, and culturally. Being compact high-level bodies, specially meant for looking after the interests of respective zones, they are capable of focusing attention on specific issues considering regional factors, while keeping the national perspective in view.

### Strengths and limitations

The implementation of favourability scores in this study serves a dual purpose in interpreting zonal disparities. Primarily, it provides a directional framework for examining zonal disparity, offering a standardised metric for comparative analysis. Moreover, these scores offer deeper insights into the performance of specific regions within zones (e.g., u_Assam, r_Assam etc.), thereby facilitating better understanding of intra-zonal dynamics (Online Resources 1, 2, and 3). This detailed perspective is particularly valuable in identifying potential resource-sharing opportunities between high-performing and underperforming regions within a zone, ultimately contributing to the holistic development of the entire zone. Further analysis revealed that all the three pillars (MHU, WE, and DC) significantly differ across the zones. Particularly for WE, only Central and East zones showed significantly poor performance as compared to the best performing South zone, while the other zones (West, North, including Northeast) have relatively similar performance. Despite this similarity, Northeast zone emerged as the worst performing zone with respect to MHU. This divergence in the relationship between WE and MHU being known to positively correlated underscores the need for a deeper examination. Further investigation indicated that WE explain only a small portion of the variability in MHU scores. This suggests that WE alone is not sufficient to improve MHU, indicating the existence of other underlying factors that impede healthcare utilisation.

### Contextual analysis of zonal healthcare disparities

The disparity in Northeast zone can be associated with this zone having the lowest full antenatal care rates due to a low number of doctors per 10,000 people, lower institutional delivery rates because of greater distances to hospitals, and low female literacy rates, which further contribute to low institutional deliveries [3]. Moreover, the lack of awareness about reproductive health, coupled with socio-economic status and cultural barriers, significantly hinders healthcare access in Northeast zone [3]. The Northeast zone, because of its geographical location, difficult terrain, high rainfall, vast hilly region, large forest areas and large number of ethnic groups, is not a very homogenous territory for easily providing normal health care services round the clock to all the 47 million people of the zone [21]. Due to inadequate communication facilities throughout the region, healthcare services have not yet become available to everyone as can normally be expected. The Government of India, in its Annual Report (2015 - 16>) has identified the following problems in the health sector in Northeast zone such as, shortage of trained manpower, providing access to sparsely populated, remote, and far-flung areas, improvement of governance in the health sector, need for improved quality of health services rendered, making effective and full utilisation of existing facilities, effective and timely utilisation of financial resources available etc. [22]. There is a concern that lower economic status could be limiting the ability to afford healthcare services and transportation, worsening disparities in access to healthcare. These factors collectively contribute to the poor maternal health outcomes. This suggests the presence of other zonal-specific challenges impacting MHU that are yet to be explored. Several factors could explain this disparity in the Northeast e.g., inadequate healthcare infrastructure, including fewer hospitals, poorly equipped facilities, and insufficient healthcare personnel. The investigators of the current study maintain that socio-geographical understanding is an equally crucial dimension which needs to be investigated. Often studies have dominantly focused on prominent proximate determinants (e.g., antenatal and postnatal care visits, skilled birth attendance etc.) of maternal health ignoring the important aspect of geographical condition as an independent factor [11]. Most of the previous studies did not account for the role of socio-geographical perspective in the maternal healthcare services accessibility especially in India [23].

### Explanation of findings

Overall, considering the existing initiatives by the Indian government such as NRHM Program started in 2005 including the RMNCHA strategy promoting institutional deliveries, ensuring skilled attendance at birth, and providing antenatal care and postnatal care later rebranded as the NHM. Two pivotal components of the NHM are the JSY and JSSK offering financial incentives and ensures that economically vulnerable pregnant women can deliver at public health facilities without incurring any costs, and infants receive free treatment for a year. These schemes could align their planning and execution processes with the findings of this study in India with particular emphasis for Northeast zone to improve MHU and DC by using the existing zonal councils and the possibility of intra-and inter- zonal council(s) co-operation restructuring by closely working with the public (implementing co-design approach, contextual analysis, understanding community system dynamics, etc.) as the study highlights that while WE is linked to better maternal healthcare outcomes, it alone does not ensure higher healthcare utilisation. The Northeast zone, despite ranking second in empowerment, has the lowest MHU, indicating that factors like accessibility, infrastructure, and socio-cultural barriers play a crucial role. The South zone’s superior performance across all pillars can be attributed to stronger public health systems, better education, and effective government schemes like JSY and JSSK. The Northeast’s challenges underline the need to address infrastructural gaps rather than solely focusing on empowerment. The favourability score framework effectively identifies underperforming regions, emphasising that a comprehensive public health strategy integrating policy, infrastructure, and socio-economic support is essential for reducing maternal health disparities.

## Limitations and future direction

Since the current study utilises NFHS data, investigators lack insights into respondents’ experiential communication with the data-gathering team. A key drawback of using SPI in this study is that investigators assumed one indicator as a single dimension, whereas SPI dimensions consist of clusters of multiple indicators. Additionally, SPI assigns weightage in its formula to ensure that different performance metrics contribute appropriately to the final score, a factor that has not been explored by the investigators.

## Conclusion

This study reveals disparities in MHU, WE, and DC across six Indian zones. Certain zones exhibit strong MHU performance despite lower WE scores, while others display unique trends, in particular, Northeast has poor MHU despite similar WE levels to better-performing zones, suggesting factors beyond WE affect MHU. It underscores the need for region-specific research and multifaceted approaches to enhance maternal healthcare access, ultimately improving outcomes for mothers and children. This study informs evidence-based strategies for better maternal healthcare.

## Supplementary Information

Supplementary Material 1.

Supplementary Material 2

## Data Availability

Organised data sets from NFHS concerning this study can be requested by contacting the corresponding author.

## Appendix A

**Table 3:** Clustering of 72 regions of 28 states and 8 UTs into six zones

States/UTs	Area	Re-coded Sample Name	Zone	States/UTs	Area	Re-coded Sample Name	Zone
India	Urban	u_India	India	Assam	Rural	r_Assam	Northeast
India	Rural	r_India	India	Manipur	Urban	u_Manipur	Northeast
Chhattisgarh	Urban	u_Chhattisgarh	Central	Manipur	Rural	r_Manipur	Northeast
Chhattisgarh	Rural	r_Chhattisgarh	Central	Meghalaya	Urban	u_Meghalaya	Northeast
Madhya Pradesh	Urban	u_Madhya Pradesh	Central	Meghalaya	Rural	r_Meghalaya	Northeast
Madhya Pradesh	Rural	r_Madhya Pradesh	Central	Mizoram	Urban	u_Mizoram	Northeast
Uttar Pradesh	Urban	u_Uttar Pradesh	Central	Mizoram	Rural	r_Mizoram	Northeast
Uttar Pradesh	Rural	r_Uttar Pradesh	Central	Nagaland	Urban	u_Nagaland	Northeast
Bihar	Urban	u_Bihar	East	Nagaland	Rural	r_Nagaland	Northeast
Bihar	Rural	r_Bihar	East	Sikkim	Urban	u_Sikkim	Northeast
Jharkhand	Urban	u_Jharkhand	East	Sikkim	Rural	r_Sikkim	Northeast
Jharkhand	Rural	r_Jharkhand	East	Tripura	Urban	u_Tripura	Northeast
Odisha	Urban	u_Odisha	East	Tripura	Rural	r_Tripura	Northeast
Odisha	Rural	r_Odisha	East	Andhra Pradesh	Urban	u_Andhra Pradesh	South
West Bengal	Urban	u_West Bengal	East	Andhra Pradesh	Rural	r_Andhra Pradesh	South
West Bengal	Rural	r_West Bengal	East	Karnataka	Urban	u_Karnataka	South
Haryana	Urban	u_Haryana	North	Karnataka	Rural	r_Karnataka	South
Haryana	Rural	r_Haryana	North	Kerala	Urban	u_Kerala	South
Himachal Pradesh	Urban	u_Himachal Pradesh	North	Kerala	Rural	r_Kerala	South
Himachal Pradesh	Rural	r_Himachal Pradesh	North	Tamil Nadu	Urban	u_Tamil Nadu	South
Jammu & Kashmir	Urban	u_Jammu & Kashmir	North	Tamil Nadu	Rural	r_Tamil Nadu	South
Jammu & Kashmir	Rural	r_Jammu & Kashmir	North	Telangana	Urban	u_Telangana	South
NCT of Delhi	Urban	u_NCT of Delhi	North	Telangana	Rural	r_Telangana	South
NCT of Delhi	Rural	r_NCT of Delhi	North	Andaman & Nicobar Islands	Urban	u_Andaman & Nicobar Islands	South
Punjab	Urban	u_Punjab	North	Andaman & Nicobar Islands	Rural	r_Andaman & Nicobar Islands	South
Punjab	Rural	r_Punjab	North	Lakshadweep	Urban	u_Lakshadweep	South
Rajasthan	Urban	u_Rajasthan	North	Lakshadweep	Rural	r_Lakshadweep	South
Rajasthan	Rural	r_Rajasthan	North	Puducherry	Urban	u_Puducherry	South
Chandigarh	Urban	u_Chandigarh	North	Puducherry	Rural	r_Puducherry	South
Chandigarh	Rural	r_Chandigarh	North	Goa	Urban	u_Goa	West
Uttarakhand	Urban	u_Uttarakhand	North	Goa	Rural	r_Goa	West
Uttarakhand	Rural	r_Uttarakhand	North	Gujarat	Urban	u_Gujarat	West
Ladakh	Urban	u_Ladakh	North	Gujarat	Rural	r_Gujarat	West
Ladakh	Rural	r_Ladakh	North	Maharashtra	Urban	u_Maharastra	West
Arunachal Pradesh	Urban	u_Arunachal Pradesh	Northeast	Maharashtra	Rural	r_Maharastra	West
Arunachal Pradesh	Rural	r_Arunachal Pradesh	Northeast	Dadra and Nagar Haveli & Daman and Diu	Urban	u_Dadra and Nagar Haveli & Daman and Diu	West
Assam	Urban	u_Assam	Northeast	Dadra and Nagar Haveli & Daman and Diu	Rural	r_Dadra and Nagar Haveli & Daman and Diu	West

## Appendix B

The pillars were selected from NFHS- 5 encompassing a range of indicators pertinent to their respective domains, called dimensions. For example, mothers who had an antenatal check-up in the first trimester, mothers who had at least four antenatal visits, mothers whose last birth was protected against neonatal tetanus, etc., are dimensions for the pillar MHU. Similarly, dimensions like currently married women who usually participate in three household decisions, women who worked in the last 12 months and were paid in cash, etc., represent the pillar WE, and dimensions like institutional births, institutional births in public facility, etc., represent the pillar DC.

**Table 4:** Pillars and dimensions of the study

Pillars	Dimensions								
MHU (for last birth in the 5 years before the survey) (%)	Mothers who had an antenatal check-up in the first trimester	Mothers who had at least antenatal care visits	Mothers whose last birth was protected against neonatal tetanus	Mothers who consumed iron folic acid for 100 days or more when they were pregnant	Mothers who consumed iron folic acid for 180 days or more when they were pregnant	Registered pregnancies for which the mother received a Mother and Child Protection (MCP) card	Mothers who received postnatal care from a doctor/nurse/LHV/ANM/midwife/other health personnel within 2 days of delivery	Children born at home who were taken to a health facility for a check-up within 24 hours of birth	Children who received postnatal care from a doctor/nurse/LHV/ANM/midwife/other health personnel within 2 days of delivery
WE (women age 15 - 49 years) (%)	Currently married women who usually participate in three household decisions	Women who worked in the last 12 months and were paid in cash	Women owning a house and/or land (alone or jointly with others)	Women having a bank or savings account that they themselves use	Women having a mobile phone that they themselves use	Women who use hygienic methods of protection during their menstrual period			
DC (for births in the 5 years before the survey) (%)	Institutional births	Institutional births in public facility	Home births that were conducted by skilled health personnel	Births attended by skilled health personnel	Births delivered by caesarean section	Births in a private health facility that were delivered by caesarean section	Births in a public health facility that were delivered by caesarean section		
