# Supplementary material for: Maternal healthcare utilisation, women empowerment, and delivery care: geographical variations in India
Source: Res Health Serv Reg. 2025 May 7;4:5. doi: 10.1007/s43999-025-00063-3 (PMC12055738; doi:10.1007/s43999-025-00063-3)
Supplement: Supplementary file 1 — Supplementary Material 1. [file 43999_2025_63_MOESM1_ESM.docx]

**Supplementary Information**

**Maternal Healthcare Utilisation, Women Empowerment, and Delivery Care: Geographical Variations in India**

Prachi Verma^1,2 [0009-0004-1972-7266]^, Ningombam Sanjib Meitei­­^3 [0000-0003-2777-5888]^, Sanjram Premjit Khanganba^1,2,4,5,6 [0000-0002-6215-7500]*^

^1^Human Factors & Applied Cognition Lab, Indian Institute of Technology Indore, Indore, 453552, India

^2^Discipline of Psychology, Indian Institute of Technology Indore, Indore, 453552, India

^3^Ningombam Angouton Memorial Trust, Imphal East, 795008, India

^4^Department of Biosciences and Biomedical Engineering, Indian Institute of Technology Indore, Indore, 453552, India

^5^Center for Electric Vehicles and Intelligent Transport SystemsIndian Institute of Technology IndoreIndore, 453552India

^6^Centre of Futuristic Defense and Space TechnologiesIndian Institute of Technology IndoreIndore, 453552India

The Quantile score range for maternal healthcare utilisation (MHU) are Quantile 1 (Q1) = 13.62- 48.08, Quantile 2 (Q2) = 48.08- 61.70, Quantile 3 (Q3) = 61.70- 75.33, and Quantile 4 (Q4) = 75.33- 88.95. The classification of regions, urban (u) and rural (r) belonging to either of the zones: East (E), West (W), North (N), South (S), Central (C), and Northeast (NE) is shown in Online Resource 1.

**Online Resource 1**

*Quantile Classification for MHU*

| Zone | Region | *SPI.PIL_ctp(MHU)_* | Quantile | Zone | Region | *SPI.PIL_ctp(MHU)_* | Quantile |
| --- | --- | --- | --- | --- | --- | --- | --- |
| N | u_Himachal Pradesh | 79.875 | Q4 | N | r_Jammu & Kashmir | 62.55556 | Q3 |
| N | r_NCT of Delhi | 78.45 | Q4 | N | u_Ladakh | 67.675 | Q3 |
| N | u_Chandigarh | 83.7375 | Q4 | N | r_Ladakh | 66.5375 | Q3 |
| NE | u_Manipur | 75.9125 | Q4 | NE | u_Assam | 63.07778 | Q3 |
| S | u_Andhra Pradesh | 80.5 | Q4 | NE | u_Mizoram | 65.7625 | Q3 |
| S | u_Kerala | 86 | Q4 | NE | r_Sikkim | 68.975 | Q3 |
| S | r_Kerala | 86.5125 | Q4 | NE | u_Tripura | 66.6625 | Q3 |
| S | u_Tamil Nadu | 86.4125 | Q4 | S | r_Andhra Pradesh | 71.4 | Q3 |
| S | r_Tamil Nadu | 85.9875 | Q4 | S | u_Karnataka | 66.12222 | Q3 |
| S | u_Telangana | 78.1125 | Q4 | S | r_Karnataka | 64.78889 | Q3 |
| S | u_Andaman & Nicobar Islands | 85.675 | Q4 | S | r_Telangana | 69.24444 | Q3 |
| S | r_Andaman & Nicobar Islands | 80.425 | Q4 | W | u_Dadra and Nagar Haveli & Daman and Diu | 75.125 | Q3 |
| S | u_Lakshadweep | 87.975 | Q4 | W | u_Gujarat | 72.11111 | Q3 |
| S | r_Lakshadweep | 82.9 | Q4 | W | r_Gujarat | 68.65556 | Q3 |
| S | u_Puducherry | 87.5 | Q4 | W | u_Maharastra | 66.2 | Q3 |
| S | r_Puducherry | 86.575 | Q4 | W | r_Maharastra | 64.41111 | Q3 |
| W | r_Dadra and Nagar Haveli & Daman and Diu | 81.4875 | Q4 | C | u_Uttar Pradesh | 57.23333 | Q2 |
| W | u_Goa | 87.425 | Q4 | C | r_Uttar Pradesh | 50.71111 | Q2 |
| W | r_Goa | 88.95 | Q4 | E | u_Bihar | 48.97778 | Q2 |
| C | u_Chhattisgarh | 63.51111 | Q3 | E | u_Jharkhand | 58.42222 | Q2 |
| C | r_Chhattisgarh | 62.06667 | Q3 | E | r_Jharkhand | 51.25556 | Q2 |
| C | u_Madhya Pradesh | 68.42222 | Q3 | N | r_Rajasthan | 59.84444 | Q2 |
| C | r_Madhya Pradesh | 63.88889 | Q3 | N | r_Uttarakhand | 59.23333 | Q2 |
| E | u_Odisha | 72.21111 | Q3 | NE | u_Arunachal Pradesh | 52.06667 | Q2 |
| E | r_Odisha | 69.81111 | Q3 | NE | r_Assam | 55.96667 | Q2 |
| E | u_West Bengal | 66.84444 | Q3 | NE | r_Manipur | 57.44444 | Q2 |
| E | r_West Bengal | 64.71111 | Q3 | NE | u_Meghalaya | 61.6375 | Q2 |
| N | u_Haryana | 67.33333 | Q3 | NE | r_Mizoram | 48.98889 | Q2 |
| N | r_Haryana | 66.82222 | Q3 | NE | u_Sikkim | 61.4125 | Q2 |
| N | r_Himachal Pradesh | 68.74444 | Q3 | NE | r_Tripura | 52.36667 | Q2 |
| N | u_NCT of Delhi | 70.64444 | Q3 | E | r_Bihar | 44.23333 | Q1 |
| N | u_Punjab | 71.15 | Q3 | NE | r_Arunachal Pradesh | 44.48889 | Q1 |
| N | r_Punjab | 65.6 | Q3 | NE | r_Meghalaya | 47.26667 | Q1 |
| N | u_Rajasthan | 70.825 | Q3 | NE | u_Nagaland | 47.93333 | Q1 |
| N | u_Uttarakhand | 66.1 | Q3 | NE | r_Nagaland | 34.45556 | Q1 |
| N | u_Jammu & Kashmir | 74.425 | Q3 |  |  |  |  |

The Quantile score range for women empowerment (WE) are, Q1= 5.86- 57.78, Q2= 57.78- 63.63, Q3= 63.63- 69.49, Q4= 69.49- 75.35. The classification is shown in Online Resource 2.

**Online Resource 2**

*Quantile Classification for WE*

| Zone | Region | SPI.PIL_ctp(WE)_ | Quantile | Zone | Region | SPI.PIL_ctp(WE)_ | Quantile |
| --- | --- | --- | --- | --- | --- | --- | --- |
| N | u_Himachal Pradesh | 71.2 | Q4 | NE | u_Mizoram | 69.05 | Q3 |
| N | u_Punjab | 70.7 | Q4 | NE | r_Mizoram | 64.26666667 | Q3 |
| N | u_Ladakh | 72.95 | Q4 | NE | u_Nagaland | 68.01666667 | Q3 |
| N | r_Ladakh | 71.11666667 | Q4 | S | u_Andhra Pradesh | 67.66666667 | Q3 |
| NE | u_Arunachal Pradesh | 73.9 | Q4 | S | r_Andhra Pradesh | 63.73333333 | Q3 |
| NE | r_Arunachal Pradesh | 70.65 | Q4 | S | r_Karnataka | 68.75 | Q3 |
| NE | u_Manipur | 72.75 | Q4 | S | u_Kerala | 67.45 | Q3 |
| NE | u_Meghalaya | 70.9 | Q4 | S | r_Kerala | 67.68333333 | Q3 |
| NE | u_Sikkim | 72.58333333 | Q4 | S | u_Andaman & Nicobar Islands | 67.73333333 | Q3 |
| NE | r_Sikkim | 69.91666667 | Q4 | S | r_Andaman & Nicobar Islands | 66.96666667 | Q3 |
| S | u_Karnataka | 72.75 | Q4 | S | u_Lakshadweep | 63.85 | Q3 |
| S | u_Tamil Nadu | 73.81666667 | Q4 | W | u_Dadra and Nagar Haveli & Daman and Diu | 69.25 | Q3 |
| S | r_Tamil Nadu | 74.96666667 | Q4 | C | r_Chhattisgarh | 59.9 | Q2 |
| S | u_Telangana | 70.73333333 | Q4 | C | u_Madhya Pradesh | 61.65 | Q2 |
| S | r_Telangana | 73.71666667 | Q4 | C | u_Uttar Pradesh | 63.5 | Q2 |
| S | u_Puducherry | 74.35 | Q4 | E | u_Bihar | 60.78333333 | Q2 |
| S | r_Puducherry | 75.35 | Q4 | E | r_Jharkhand | 61.38333333 | Q2 |
| W | r_Dadra and Nagar Haveli & Daman and Diu | 69.5 | Q4 | E | r_Odisha | 62.71666667 | Q2 |
| W | u_Goa | 70.45 | Q4 | N | r_Haryana | 58.6 | Q2 |
| W | r_Goa | 71.28333333 | Q4 | N | u_Rajasthan | 62.25 | Q2 |
| C | u_Chhattisgarh | 65.55 | Q3 | N | r_Uttarakhand | 60.51666667 | Q2 |
| E | u_Jharkhand | 67.23333333 | Q3 | NE | r_Assam | 58.43333333 | Q2 |
| E | u_Odisha | 64.15 | Q3 | NE | r_Nagaland | 59.61666667 | Q2 |
| E | u_West Bengal | 64.5 | Q3 | NE | u_Tripura | 58.66666667 | Q2 |
| N | u_Haryana | 64.51666667 | Q3 | S | r_Lakshadweep | 57.88 | Q2 |
| N | r_Himachal Pradesh | 64.3 | Q3 | W | u_Gujarat | 63.25 | Q2 |
| N | u_NCT of Delhi | 63.75 | Q3 | W | u_Maharastra | 62.38333333 | Q2 |
| N | r_NCT of Delhi | 67.18333333 | Q3 | W | r_Maharastra | 57.9 | Q2 |
| N | r_Punjab | 67.75 | Q3 | C | r_Madhya Pradesh | 51.91666667 | Q1 |
| N | u_Uttarakhand | 64.03333333 | Q3 | C | r_Uttar Pradesh | 56.66666667 | Q1 |
| N | u_Chandigarh | 66.23333333 | Q3 | E | r_Bihar | 56.16666667 | Q1 |
| N | u_Jammu & Kashmir | 67.05 | Q3 | E | r_West Bengal | 53.41666667 | Q1 |
| N | r_Jammu & Kashmir | 64.56666667 | Q3 | N | r_Rajasthan | 56.18333333 | Q1 |
| NE | u_Assam | 64.6 | Q3 | NE | r_Tripura | 53.7 | Q1 |
| NE | r_Manipur | 69.43333333 | Q3 | W | r_Gujarat | 55.06666667 | Q1 |
| NE | r_Meghalaya | 65.46666667 | Q3 |  |  |  |  |

The Quantile score range for delivery care (DC) are, Q1= 11.71- 36.05, Q2 = 36.05- 47.76, Q3= 47.76- 59.47, and Q4= 59.47- 71.18. The classification is shown in Online Resource 3.

**Online Resource 3**

*Quantile Classification for DC*

| Zone | Region | *SPI.PIL_ctp(WE)_* | Quantile | Zone | Region | *SPI.PIL_ctp(WE)_* | Quantile |
| --- | --- | --- | --- | --- | --- | --- | --- |
| N | u_Chandigarh | 64.08333 | Q4 | S | r_Karnataka | 52.15714 | Q3 |
| N | u_Jammu & Kashmir | 68.85714 | Q4 | S | u_Kerala | 49.6 | Q3 |
| N | r_Jammu & Kashmir | 61.04286 | Q4 | S | r_Kerala | 50.41429 | Q3 |
| N | u_Ladakh | 65.65 | Q4 | S | u_Tamil Nadu | 57.78571 | Q3 |
| N | r_Ladakh | 59.75 | Q4 | S | r_Andaman & Nicobar Islands | 54.16667 | Q3 |
| NE | u_Tripura | 65 | Q4 | S | u_Lakshadweep | 49.97143 | Q3 |
| S | r_Tamil Nadu | 59.72857 | Q4 | S | u_Puducherry | 55.42857 | Q3 |
| S | u_Telangana | 61.17143 | Q4 | W | u_Dadra and Nagar Haveli & Daman and Diu | 50.3 | Q3 |
| S | r_Telangana | 61.15714 | Q4 | W | r_Dadra and Nagar Haveli & Daman and Diu | 49.52857 | Q3 |
| S | u_Andaman & Nicobar Islands | 70.42 | Q4 | W | u_Maharastra | 48.45714 | Q3 |
| S | r_Lakshadweep | 71.18 | Q4 | C | r_Chhattisgarh | 46.17143 | Q2 |
| S | r_Puducherry | 68.18333 | Q4 | C | r_Madhya Pradesh | 47.37143 | Q2 |
| W | u_Goa | 61.65 | Q4 | C | u_Uttar Pradesh | 43.34286 | Q2 |
| W | r_Goa | 64.56667 | Q4 | C | r_Uttar Pradesh | 40.9 | Q2 |
| C | u_Chhattisgarh | 51.41429 | Q3 | E | u_Bihar | 39.32857 | Q2 |
| C | u_Madhya Pradesh | 50.2 | Q3 | E | r_Bihar | 38.71429 | Q2 |
| E | u_Odisha | 55.58571 | Q3 | E | u_Jharkhand | 45.58571 | Q2 |
| E | r_Odisha | 52.84286 | Q3 | E | r_Jharkhand | 40.58571 | Q2 |
| E | u_West Bengal | 58.34286 | Q3 | N | u_Haryana | 44.81429 | Q2 |
| E | r_West Bengal | 56.71429 | Q3 | N | r_Haryana | 44.7 | Q2 |
| N | u_Himachal Pradesh | 48.72857 | Q3 | N | u_NCT of Delhi | 47.75714 | Q2 |
| N | r_Himachal Pradesh | 48.4 | Q3 | N | r_NCT of Delhi | 46.72857 | Q2 |
| N | u_Punjab | 51.32857 | Q3 | N | r_Rajasthan | 43.91429 | Q2 |
| N | r_Punjab | 53.78571 | Q3 | N | u_Uttarakhand | 46.65714 | Q2 |
| N | u_Rajasthan | 47.82857 | Q3 | N | r_Uttarakhand | 41.32857 | Q2 |
| NE | u_Arunachal Pradesh | 51.04286 | Q3 | NE | r_Arunachal Pradesh | 44.41429 | Q2 |
| NE | u_Assam | 57.37143 | Q3 | NE | r_Manipur | 43.95714 | Q2 |
| NE | r_Assam | 48.91429 | Q3 | NE | u_Meghalaya | 44.64286 | Q2 |
| NE | u_Manipur | 55.05714 | Q3 | NE | r_Mizoram | 37.44286 | Q2 |
| NE | u_Mizoram | 48.45714 | Q3 | W | u_Gujarat | 45.9 | Q2 |
| NE | u_Sikkim | 56.6 | Q3 | W | r_Gujarat | 40.22857 | Q2 |
| NE | r_Sikkim | 54.11429 | Q3 | W | r_Maharastra | 45.9 | Q2 |
| NE | r_Tripura | 49.34286 | Q3 | NE | r_Meghalaya | 31.35714 | Q1 |
| S | u_Andhra Pradesh | 55.31429 | Q3 | NE | u_Nagaland | 33.64286 | Q1 |
| S | r_Andhra Pradesh | 53.12857 | Q3 | NE | r_Nagaland | 24.34286 | Q1 |
| S | u_Karnataka | 51.77143 | Q3 |  |  |  |  |

Online Resource 4 shows Multivariate analysis of variance (MANOVA) result, it revealed significant differences across the zones for all three pillars (MHU, WE, and DC), as indicated by the following *F*-values and *p*-values: MHU: *F* (5, 65) = 15.463, *p* < .001, WE: *F* (5, 65) = 4.175, *p* < .002, and DC: *F* (5, 65) = 3.316, *p* < .010.

**Online Resource 4**

*MANOVA For Pillars*

| Source | Dependent Variable | Type III Sum of Squares | df | Mean Square | F | Sig. |
| --- | --- | --- | --- | --- | --- | --- |
| Corrected Model | MHU | 5912.660^a^ | 5 | 1182.532 | 15.463 | <.001 |
|  | WE | 567.836^b^ | 5 | 113.567 | 4.175 | .002 |
|  | DC | 1194.693^c^ | 5 | 238.939 | 3.316 | .010 |
| Intercept | MHU | 269617.042 | 1 | 269617.042 | 3525.489 | <.001 |
|  | WE | 249529.737 | 1 | 249529.737 | 9173.568 | <.001 |
|  | DC | 152013.507 | 1 | 152013.507 | 2109.571 | <.001 |
| Zones | MHU | 5912.660 | 5 | 1182.532 | 15.463 | <.001 |
|  | WE | 567.836 | 5 | 113.567 | 4.175 | .002 |
|  | DC | 1194.693 | 5 | 238.939 | 3.316 | .010 |
| Error | MHU | 4970.973 | 65 | 76.477 |  |  |
|  | WE | 1768.062 | 65 | 27.201 |  |  |
|  | DC | 4683.833 | 65 | 72.059 |  |  |
| Total | MHU | 337661.676 | 71 |  |  |  |
|  | WE | 306991.246 | 71 |  |  |  |
|  | DC | 191086.873 | 71 |  |  |  |
| Corrected Total | MHU | 10883.632 | 70 |  |  |  |
|  | WE | 2335.898 | 70 |  |  |  |
|  | DC | 5878.525 | 70 |  |  |  |

*Note:* a= R Squared = .543 (Adjusted R Squared = .508), b= R Squared = .243 (Adjusted R Squared = .185), and c= R Squared = .203 (Adjusted R Squared = .142) Online Resource 5 shows Bonferroni post-hoc test, several significant mean differences were found. For MHU, the C zone differed significantly from the S zone (-19.41, *p* < .002), C vs W (-14.57, *p* < .045) E vs. S (-20.82, *p* < .001), E vs. W (-15.98 , *p* < .09), N vs S (-10.99, *p* < .009 ), NE vs. S (-23.85, *p* < 0.001), NE vs. W (-19.01, *p* < .001), and NE vs. N (-12.85, *p* < .001). For WE, significant differences were observed between the C and S zones (-9.34, *p* < .006) and E vs. S (-7.91, *p* < .012). For DC, the NE zone differed significantly from the S zone (-10.99, *p* < 0.008).

**Online Resource 5**

*Bonferroni* *Multiple Comparisons Post Hoc Test for Pillars*

| Dependent Variable | Zone | | Mean Difference (I-J) | Std. Error | Sig. | 95% Confidence Interval | |
| --- | --- | --- | --- | --- | --- | --- | --- |
|  |  |  |  |  |  | Lower Bound | Upper Bound |
| MHU | C | East | 1.4139 | 4.72289 | 1.000 | -12.9794 | 15.8071 |
|  |  | North | -8.4132 | 4.15267 | .703 | -21.0687 | 4.2423 |
|  |  | North-East | 4.4461 | 4.18639 | 1.000 | -8.3122 | 17.2043 |
|  |  | South | -19.4109* | 4.18639 | <.001 | -32.1692 | -6.6527 |
|  |  | West | -14.5734* | 4.72289 | .045 | -28.9667 | -.1802 |
|  | E | Central | -1.4139 | 4.72289 | 1.000 | -15.8071 | 12.9794 |
|  |  | North | -9.8271 | 3.74942 | .164 | -21.2537 | 1.5994 |
|  |  | North-East | 3.0322 | 3.78673 | 1.000 | -8.5081 | 14.5725 |
|  |  | South | -20.8248* | 3.78673 | <.001 | -32.3651 | -9.2846 |
|  |  | West | -15.9873* | 4.37254 | .008 | -29.3129 | -2.6618 |
|  | N | Central | 8.4132 | 4.15267 | .703 | -4.2423 | 21.0687 |
|  |  | East | 9.8271 | 3.74942 | .164 | -1.5994 | 21.2537 |
|  |  | North-East | 12.8593* | 3.04605 | .001 | 3.5763 | 22.1423 |
|  |  | South | -10.9977* | 3.04605 | .009 | -20.2807 | -1.7147 |
|  |  | West | -6.1602 | 3.74942 | 1.000 | -17.5868 | 5.2664 |
|  | NE | Central | -4.4461 | 4.18639 | 1.000 | -17.2043 | 8.3122 |
|  |  | East | -3.0322 | 3.78673 | 1.000 | -14.5725 | 8.5081 |
|  |  | North | -12.8593* | 3.04605 | .001 | -22.1423 | -3.5763 |
|  |  | South | -23.8570* | 3.09185 | <.001 | -33.2796 | -14.4344 |
|  |  | West | -19.0195* | 3.78673 | <.001 | -30.5598 | -7.4793 |
|  | S | Central | 19.4109* | 4.18639 | <.001 | 6.6527 | 32.1692 |
|  |  | East | 20.8248* | 3.78673 | <.001 | 9.2846 | 32.3651 |
|  |  | North | 10.9977* | 3.04605 | .009 | 1.7147 | 20.2807 |
|  |  | North-East | 23.8570* | 3.09185 | <.001 | 14.4344 | 33.2796 |
|  |  | West | 4.8375 | 3.78673 | 1.000 | -6.7028 | 16.3778 |
|  | W | Central | 14.5734* | 4.72289 | .045 | .1802 | 28.9667 |
|  |  | East | 15.9873* | 4.37254 | .008 | 2.6618 | 29.3129 |
|  |  | North | 6.1602 | 3.74942 | 1.000 | -5.2664 | 17.5868 |
|  |  | North-East | 19.0195* | 3.78673 | <.001 | 7.4793 | 30.5598 |
|  |  | South | -4.8375 | 3.78673 | 1.000 | -16.3778 | 6.7028 |
| WE | C | East | -1.4299 | 2.81667 | 1.000 | -10.0138 | 7.1541 |
|  |  | North | -5.6008 | 2.47660 | .406 | -13.1484 | 1.9468 |
|  |  | North-East | -6.5080 | 2.49671 | .170 | -14.1168 | 1.1009 |
|  |  | South | -9.3484* | 2.49671 | .006 | -16.9573 | -1.7396 |
|  |  | West | -5.0215 | 2.81667 | 1.000 | -13.6055 | 3.5624 |
|  | E | Central | 1.4299 | 2.81667 | 1.000 | -7.1541 | 10.0138 |
|  |  | North | -4.1710 | 2.23611 | 1.000 | -10.9856 | 2.6437 |
|  |  | North-East | -5.0781 | 2.25836 | .419 | -11.9606 | 1.8043 |
|  |  | South | -7.9185* | 2.25836 | .012 | -14.8010 | -1.0361 |
|  |  | West | -3.5917 | 2.60773 | 1.000 | -11.5389 | 4.3555 |
|  | N | Central | 5.6008 | 2.47660 | .406 | -1.9468 | 13.1484 |
|  |  | East | 4.1710 | 2.23611 | 1.000 | -2.6437 | 10.9856 |
|  |  | North-East | -.9072 | 1.81662 | 1.000 | -6.4434 | 4.6291 |
|  |  | South | -3.7476 | 1.81662 | .647 | -9.2838 | 1.7887 |
|  |  | West | .5793 | 2.23611 | 1.000 | -6.2354 | 7.3939 |
|  | NE | Central | 6.5080 | 2.49671 | .170 | -1.1009 | 14.1168 |
|  |  | East | 5.0781 | 2.25836 | .419 | -1.8043 | 11.9606 |
|  |  | North | .9072 | 1.81662 | 1.000 | -4.6291 | 6.4434 |
|  |  | South | -2.8404 | 1.84394 | 1.000 | -8.4599 | 2.7791 |
|  |  | West | 1.4865 | 2.25836 | 1.000 | -5.3960 | 8.3689 |
|  | S | Central | 9.3484* | 2.49671 | .006 | 1.7396 | 16.9573 |
|  |  | East | 7.9185* | 2.25836 | .012 | 1.0361 | 14.8010 |
|  |  | North | 3.7476 | 1.81662 | .647 | -1.7887 | 9.2838 |
|  |  | North-East | 2.8404 | 1.84394 | 1.000 | -2.7791 | 8.4599 |
|  |  | West | 4.3269 | 2.25836 | .897 | -2.5556 | 11.2093 |
|  | W | Central | 5.0215 | 2.81667 | 1.000 | -3.5624 | 13.6055 |
|  |  | East | 3.5917 | 2.60773 | 1.000 | -4.3555 | 11.5389 |
|  |  | North | -.5793 | 2.23611 | 1.000 | -7.3939 | 6.2354 |
|  |  | North-East | -1.4865 | 2.25836 | 1.000 | -8.3689 | 5.3960 |
|  |  | South | -4.3269 | 2.25836 | .897 | -11.2093 | 2.5556 |
| DC | C | East | -1.8958 | 4.58445 | 1.000 | -15.8672 | 12.0755 |
|  |  | North | -5.5130 | 4.03095 | 1.000 | -17.7976 | 6.7715 |
|  |  | North-East | -.0396 | 4.06368 | 1.000 | -12.4239 | 12.3447 |
|  |  | South | -11.0320 | 4.06368 | .127 | -23.4163 | 1.3523 |
|  |  | West | -4.2497 | 4.58445 | 1.000 | -18.2211 | 9.7217 |
|  | E | Central | 1.8958 | 4.58445 | 1.000 | -12.0755 | 15.8672 |
|  |  | North | -3.6172 | 3.63952 | 1.000 | -14.7088 | 7.4744 |
|  |  | North-East | 1.8563 | 3.67574 | 1.000 | -9.3458 | 13.0583 |
|  |  | South | -9.1362 | 3.67574 | .233 | -20.3382 | 2.0658 |
|  |  | West | -2.3539 | 4.24438 | 1.000 | -15.2888 | 10.5811 |
|  | N | Central | 5.5130 | 4.03095 | 1.000 | -6.7715 | 17.7976 |
|  |  | East | 3.6172 | 3.63952 | 1.000 | -7.4744 | 14.7088 |
|  |  | North-East | 5.4734 | 2.95676 | 1.000 | -3.5375 | 14.4843 |
|  |  | South | -5.5190 | 2.95676 | .997 | -14.5299 | 3.4919 |
|  |  | West | 1.2633 | 3.63952 | 1.000 | -9.8283 | 12.3550 |
|  | NE | Central | .0396 | 4.06368 | 1.000 | -12.3447 | 12.4239 |
|  |  | East | -1.8563 | 3.67574 | 1.000 | -13.0583 | 9.3458 |
|  |  | North | -5.4734 | 2.95676 | 1.000 | -14.4843 | 3.5375 |
|  |  | South | -10.9924* | 3.00123 | .008 | -20.1388 | -1.8460 |
|  |  | West | -4.2101 | 3.67574 | 1.000 | -15.4121 | 6.9919 |
|  | S | Central | 11.0320 | 4.06368 | .127 | -1.3523 | 23.4163 |
|  |  | East | 9.1362 | 3.67574 | .233 | -2.0658 | 20.3382 |
|  |  | North | 5.5190 | 2.95676 | .997 | -3.4919 | 14.5299 |
|  |  | North-East | 10.9924* | 3.00123 | .008 | 1.8460 | 20.1388 |
|  |  | West | 6.7823 | 3.67574 | 1.000 | -4.4197 | 17.9843 |
|  | W | Central | 4.2497 | 4.58445 | 1.000 | -9.7217 | 18.2211 |
|  |  | East | 2.3539 | 4.24438 | 1.000 | -10.5811 | 15.2888 |
|  |  | North | -1.2633 | 3.63952 | 1.000 | -12.3550 | 9.8283 |
|  |  | North-East | 4.2101 | 3.67574 | 1.000 | -6.9919 | 15.4121 |
|  |  | South | -6.7823 | 3.67574 | 1.000 | -17.9843 | 4.4197 |

*Note:* * = The mean difference is significant at the .05 level and the error term is Mean Square (Error) = 72.059

Online Resource 6 gives information about coefficient of determination (*R^2^)*. The model shows less than 16% variability in MHU. The correlation is between WE and MHU is significant, *p* < .001. The regression coefficient shows WE is a significant predictor with a positive relationship to MHU.

**Online Resource 6**

*Univariate Linear Regression Output for MHU with WE*

| Variable | R Square | Unstandardised coefficient B | Sig. | Pearson Correlation | Sig. (1-tailed) |
| --- | --- | --- | --- | --- | --- |
| WE | .166 | .880 | <.001 | .408 | <.001 |

Note: Predictor: WE
